# Multidrug-Resistant Virulent *Escherichia coli* Strains in Cattle: Implications on Food Safety and Public Health

**DOI:** 10.1155/ijm/9956724

**Published:** 2025-09-03

**Authors:** Ajay Kumar, Emmanuel W. Bumunang, Peter Kotsoana Montso, Smriti Singh, Collins Njie Ateba

**Affiliations:** ^1^Department of Industrial Microbiology, Jacob Institute of Biotechnology and Bioengineering, Sam Higginbottom University of Agriculture, Technology and Sciences, Prayagraj, Uttar Pradesh, India; ^2^Food Security and Safety Niche Area, Faculty of Agriculture, Science and Technology, North West University, Mmabatho, Mafikeng, South Africa; ^3^Department of Biological Sciences, University of Lethbridge, Lethbridge, Alberta, Canada; ^4^The School for Data Science and Computational Thinking, Stellenbosch University, Stellenbosch, South Africa; ^5^Department of Microbiology, Faculty of Science, Stellenbosch University, Stellenbosch, South Africa; ^6^Department of Anaesthesia and Operation Theatre Technology, Chandigarh Pharmacy College, Chandigarh Group of Colleges, Mohali, Punjab, India; ^7^School of Biology and Environmental Sciences, Faculty of Agriculture and Natural Sciences, University of Mpumalanga, Mbombela, South Africa

**Keywords:** biofilm, cattle, *Escherichia coli*, genetic diversity, multiple antibiotic resistance, PFGE, virulence genes

## Abstract

*Escherichia coli* inhabit the gastrointestinal tract of mammals, including cattle, where they occur as commensals. However, some strains have evolved as highly virulent pathogens that also harbor a variety of multidrug-resistant determinants. In the present study, from cattle fecal samples, a total of 636 confirmed *E. coli* strains were obtained based on the presence of the *uidA* housekeeping gene. Of the seven antibiotics tested, 120 isolates displayed multiple antibiotic-resistant (MAR) traits, with two strains (ERO138 and EKL68) showing resistance to six antibiotics. The *hlyA* (62.5%) was the most prevalent among the MAR isolates. In addition, 11 (9.1%) isolates harbored all four screened virulence genes (*eaeA*, *stx1*, *stx2*, and *hlyA*). Seven of the 120 MAR isolates displayed moderate biofilm-producing properties, and two of these isolates (ERO157 and EKL127) harbored all four virulence genes tested. Pulsed field gel electrophoresis (PFGE) analysis revealed that all 120 MAR isolates clustered into eight groups, displaying high genetic variability. These findings are important for screening and monitoring of diverse *E. coli* isolates from cattle in the Northwest region of South that harbors virulence and multiple antibiotic resistance traits.

## 1. Introduction

The environment is a rich repository of diversity of microbes (pathogenic, nonpathogenic, and opportunistic pathogens), which are continuously exchanging genetic material between different ecological niches through various carriers/hosts. *Escherichia coli* occurs as normal flora in the gastrointestinal tract of warm-blooded animals, including cattle, where it establishes a mutualistic relationship with the host [1, 2]. However, some strains have acquired virulence genes, which are implicated in a variety of human and animal diseases [3–5]. Pathogenic *E. coli* is classified into extraintestinal pathogenic *E. coli* (ExPEC) and intestinal pathogenic *E. coli* (InPEC). The ExPEC strains cause diseases exterior to the intestine, such as septicemia, meningitis, and urinary tract infections (UTIs) while InPEC strains are associated with diarrheagenic infections. Based on the diseases and clinical symptoms or manifestations, diarrheagenic *E. coli* (DEC) are classified into different pathotypes that comprise enterohemorrhagic (shiga-toxin producing) *E. coli* (EHEC/STEC), enteropathogenic *E. coli* (EPEC), enteroinvasive *E. coli* (EIEC), enteroaggregative *E. coli* (EAEC), diffusely adherent *E. coli* (DEAC), adherent invasive *E. coli* (AIEC), and enterotoxigenic *E. coli* (ETEC) [1, 3, 5].

The STEC pathotype has received significant attention and is considered an important group with significant public health concern. STEC strains have the ability to cause hemolytic uremic syndrome (HUS), hemorrhagic colitis, and thrombotic thrombocytopenic purpura that present severe health challenges, including death in patients [6, 7]. Notably, STEC strains have a very low (less than 50 bacterial cells) infection dose; thus, their severe public health implications [6]. In addition, STECs harbor a variety of virulence determinants, with the shiga-toxins (stxs) considered the principal genes. To date, two main types, *stx_1_* and *stx_2_*, have been identified and classified. Other subtypes of *stx_1_* (*stx_1a_*, *stx_1c_*, and *stx_1d_*) and *stx_2_* (*stx_2a_*, *stx_2b_*, *stx_2c_*, *stx_2d_*, *stx_2e_*, *stx_2f_,* and *stx_2g_*) have also been reported in STEC strains [7, 8]. The virulence determinants *eaeA* encode the intimin protein that is responsible for initiating attachment and effacing lesions on intestinal mucosal cells [9] while the plasmid-encoded *hlyA* facilitates hemolysis by producing cytolysin, which exacerbates the aftermath of an infection coupled with a rampant increase in resistance to most antibiotics.

Findings from several studies reveal an increasing trend in antibiotic resistance, particularly among foodborne pathogens resulting mainly from the misuse of antibiotics in agriculture and human medicine [10–12]. The impact of antibiotic-resistant strains is compounded by the potential to also display biofilm formation. Biofilms are a complex group of microbial cells that adhere to the exopolysaccharide matrix, and this increases the antibiotic resistance potential of the organisms due to (i) limited diffusion of antimicrobial agents through the biofilm matrix, (ii) communication of the antimicrobial agents with the biofilm matrix (polymer and cells), (iii) enzyme-mediated resistance, and (iv) levels of metabolic activity within the biofilm [13]. The altered environment with the biofilm results in an alteration of the growth kinetics of bacteria, making it more challenging to treat infections caused by these strains [14]. However, there is very little progress in the development of new antibiotics; thus, antibiotic resistance remains a severe public health concern globally.

*E. coli* strains constantly evolve and display genetic heterogeneity due to horizontal gene transfer (HGT), recombination, and mutations [15–17]. This genetic heterogeneity may produce clonal strains that may be very difficult to differentiate, especially during outbreaks. Molecular characterization and typing of these pathogens at the genetic level is eminent. Molecular typing may generate highly useful epidemiological data that may assist in point source determination and urgent prevention of cross-contamination [18]. Among the bacterial typing techniques, pulsed-field gel electrophoresis (PFGE) is considered a gold standard technique to determine the genetic similarities and diversity among highly related strains [19, 20]. PFGE provides high-resolution separation of DNA fragments from kilobase to megabase lengths, making it an effective method for detailed bacterial strain typing and determining clonal relationships [21, 22]. Classification of strains is achieved by analyzing distinct pulsotypes, which are defined by the specific number and size of DNA bands detected through gel electrophoresis [22, 23]. This study is aimed at determining the virulence determinants, antibiotic resistance, and genetic diversity of *E. coli* strains circulating within cattle in the Northwest Province, South Africa.

## 2. Materials and Methods

### 2.1. Ethical Clearance

Prior to the collection of samples, ethical clearance (NWU-00066-15-S9) was obtained from the Research Ethics Committee of North West University-Mafikeng Campus, South Africa.

### 2.2. Sample Collection and Isolation of *E. coli*

Commercial farms located in Northwest Province (South Africa) were targeted for the collection of cattle fecal samples (Table 1). Fecal samples were collected directly from the rectum of animals using sterile arm-length gloves and placed into sterile containers. The samples were transported on ice to the laboratory for microbiological analysis. Then, 1 g of each fecal sample was dissolved in 1% (*w*/*v*) buffered peptone water, and 10-fold serial dilutions were prepared. Aliquots of 100 *μ*L of dilutions 10^−5^ were spread-plated on Sorbitol MacConkey (SMAC) agar (BioLab, South Africa). The SMAC plates were incubated aerobically at 37°C for 24 h. After incubation, isolates that demonstrated characters of *E. coli* (pink and nonpink colonies) were selected and purified by subculturing on SMAC plates. The plates were incubated aerobically at 37°C for 24 h. Pure colonies were stored at 4°C for further microbiological analysis.

### 2.3. Molecular Identification of Isolates

Genomic DNA was extracted from the isolates using the cetyltrimethylammonium bromide (CTAB) method by Wilson [24]. The gDNA was used to perform PCR to confirm the identity of *E. coli* isolates by targeting genus-specific *uidA* housekeeping gene [25]. Standard 25-*μ*L PCR reactions consisted of 12.5 *μ*L of One Taq Quick-Load 2X Master mix (New England Biolabs), 11 *μ*L of nuclease-free water, 0.25 *μ*L of each primer (10 *μ*M), and 1 *μ*L of DNA template. The oligonucleotide primer sequences and PCR conditions used appear in Table 2. The PCR products were resolved by electrophoresis on a 1.5% (*w*/*v*) agarose gel containing ethidium bromide (0.1 *μ*g/mL) at 60 V for 1 hr. A 100 bp DNA ladder (New England Biolabs, United Kingdom) was utilized to confirm the amplicon size of the amplified gene. The images were captured in the ChemiDoc Imaging System (Bio-RAD ChemiDoc MP Imaging System, United Kingdom) using Gene Snap (Version 6.00.22) software.

### 2.4. Antibiotic Susceptibility Test

The disc diffusion technique was used to determine the resistance profiles of all the confirmed *E*. *coli* isolates against a panel of seven antibiotics following the Clinical Laboratory Standard Institute (CLSI) guidelines [27]. The antibiotics tested were gentamicin (10 *μ*g), ertapenem (10 *μ*g), ampicillin (AMP) (10 *μ*g), tetracycline (30 *μ*g), cefotaxime (CTX) (30 *μ*g), norfloxacin (NOR) (10 *μ*g,) and chloramphenicol (30 *μ*g). Isolates were cultured in tryptic soy broth (TSB) and 100 *μ*L of exponential phase competent cells (equivalent to a 0.5 MacFarland's solution) were spread-plated on Mueller–Hinton agar plates. The antibiotic discs were placed on plates at equal distances. The plates were incubated aerobically at 37°C for 24 h. After incubation, growth inhibition zone diameters were measured in mm. The data were recorded, and the results were interpreted using CLSI guidelines. Isolates that were resistant to three or more antibiotics were considered MAR strains and were selected for further microbiological analysis.

### 2.5. PCR Profiling of Virulence Determinants

The MAR isolates were subjected to multiplex PCR for profiling of virulence genes (*stx*_1_, *stx*_2_, and *hlyA*) whereas the *eaeA* gene was screened using a monoplex PCR [26]. Details of the oligonucleotide primer sequences used and PCR conditions are shown in Table 2. The PCR mixture constituted 12.5 *μ*L of One Taq Quick-Load 2X Master mix (New England Biolabs, United Kingdom), 0.25 *μ*L of each primer (30 *μ*M), 1 *μ*L of DNA template, and nuclease-free water added to make a final volume of 25 *μ*l. The amplified PCR products were separated by electrophoresis on a 1.5% (*w*/*v*) agarose gel.

### 2.6. Biofilm Formation

The MAR isolates (*n* = 120) were further screened for their potential to form biofilms. To achieve this, the microtitre plate assay was performed in triplicate as described earlier by Wang et al. [28] with some modification. The test isolates were grown to exponential phase cells in Luria Bertani (LB) broth (Biolab, SA) at 37°C. After incubation, a 1:100 dilution of the bacterial cultures was prepared using sterile LB broth. Then, 200 *μ*L of diluted cultures were dispensed into the wells of the microtitre plates, and the plates were incubated aerobically at 37°C for 24 h. Wells containing sterile uninoculated LB broth were used as negative controls. After incubation, the LB broth and cultures in the wells of the plates were discarded. The wells were washed twice with phosphate buffer saline (PBS) to remove unattached bacterial cells. After washing, 200 *μ*L of 1% (*w*/*v*) crystal violet solution was dispensed in each well, and the plates were kept at room temperature for 1 h. The dye was discarded, and the wells were washed five times with PBS. The plates were allowed to air-dry at room temperature, and 200 *μ*L of 95% (*v*/*v*) ethanol was dispensed into each well. The plates were held at room temperature for 5 mins. The ethanol-containing extracted dye was transferred into the wells of new microtitre plates, and OD was measured at 595 nm using a Microtitre Plate Reader (BioTek Instruments, Inc., Winooski, VT, United States). The biofilm forming potentials of the isolates were classified as nonbiofilm formers when OD < OD_C_, weak biofilm formers when OD_C_ < OD<2OD_C_), moderate biofilm formers when 2OD_C_ < OD < 4OD_C_, and strong biofilm formers when 4OD_C_ < OD.

### 2.7. Molecular Typing of *E. coli* Using PFGE

The genetic relatedness of the *E. coli* isolates was determined using PFGE as described in the PulseNet standardized protocol for *E. coli* O157:H7 and non-O157 [29]. The restriction enzyme *XbaI* (New England Biolabs, United Kingdom) was used to digest the DNA in prepared agarose plugs, and the image of the final gel was captured in a ChemiDoc Imaging System (Bio-RAD ChemiDoc MP Imaging System, United Kingdom). Electrophoretic gel bands were scored using GeneTools (Syngene, Synoptics, United Kingdom) and used to construct a band present/absence (0/1) matrix. This matrix was used for cluster analysis with Statistical Version 10.0 (Statsoft, United States) based on the Wards algorithm and Euclidean distance.

## 3. Results

### 3.1. Isolation and Identification of *E. coli* From Fecal Samples

**Figure 1:**
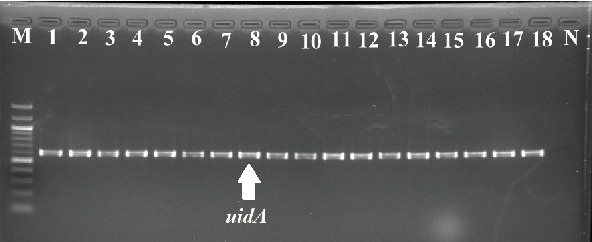
PCR amplification of *E. coli* genus-specific *uidA* gene (556 bp). Lane M is 100 bp ladder; N is negative control, and Lanes 1–18 are some of the representative *E. coli* strains isolated from cattle fecal samples.

A total of 1566 presumptive isolates were obtained from 261 fecal samples. PCR amplification results showed that 40.6% of the isolates were *E. coli* (Figure 1). Of the confirmed (636) *E. coli* isolates, 160, 181, 127, and 168 isolates were from the samples obtained from commercial farms in Rooigrond (RO), Zwartfontein (ZW), Koster (KO), and Klippan (KL), respectively.

### 3.2. Antibacterial Susceptibility Test

The susceptibility test results showed that out of 636 isolates, 528 isolates (83.01%) were resistant to CTX followed by 414 isolates (65.09%) that were resistant to AMP. On the other hand, 39 (6.13%), 19 (2.98%), 25 (3.93%), and 128 (20.12%) isolates were found resistant to ertapenem, chloramphenicol, gentamicin, and tetracycline, respectively, whereas all the tested strains were found susceptible to NOR. Of the 636 isolates tested, 120 (18.86%) isolates (RO, *n* = 58; ZW, *n* = 22; KL, *n* = 19; and KO, *n* = 21) showed a multiple antibiotic resistant (MAR) profile. Out of 58 MAR strains isolated from RO commercial farm, 36 strains were resistant to 3 antibiotics, 16 strains were resistant to 4 antibiotics, 5 strains were resistant to 5 antibiotics (ERO20, ERO77, ERO92, ERO94, and ERO100), and 1 isolate (ERO138) showed resistance to six antibiotics. Of the 22 MAR isolates from ZW commercial farm, 18 strains showed resistance to three antibiotics and 3 strains showed resistance to 4 antibiotics (EZW9, EZW24, and EZW31), whereas 1 isolate (EZW26) showed resistance to 5 antibiotics. From KL commercial farm, 19 MAR isolates were isolated, of which 18 were resistant to 3 antibiotics, and 1 isolate (EKL68) showed resistance to six antibiotics. A total of 21 MAR strains from KO Commercial farm were isolated, of which 20 strains showed resistance to 3 antibiotics, whereas 1 isolate (EKO86) showed resistance to 4 antibiotics. The common MAR phenotype that prevails in different commercial farms was CTX-AMP-TE.

### 3.3. Profiling of Virulence Genes

In the present study, virulence determinants (*stx1*, *stx2*, *eaeA*, and *hlyA*) were screened in 120 MAR isolates. The PCR results showed the existence of four screened virulence determinants in the strains obtained from different locations (Table 3). The PCR results confirmed the presence of screened virulence genes (≤ 1 genes) in 110 strains, whereas 10 strains showed no virulence determinants. The virulence gene most commonly circulating in all the sampled areas was *hlyA*, which was harbored by 75 (62.5%) MAR isolates. After the hlyA gene, the *stx2* virulence gene (61 isolates/50.8%) was more prevalent, followed by the *stx1* gene (45 isolates/37.5%) whereas the *eaeA* gene (32 isolates/26.6%) was the least prevalent in the studied MAR isolates. Out of 120 tested MAR isolates, 11 isolates (9.1%) viz., ERO2, ERO45, ERO54, ERO92, ERO157, EZO106, EKO17, EKO52, EKO118, EKL68, and EKL127 harbored all four screened virulence genes (*stx1, stx2, eaeA*, and hlyA). On the other hand, three isolates (2.5%) harbored three virulence genes viz., *stx2, hlyA,* and *eaeA,* whereas three isolates (2.5%) viz., ERO48, EZO179, and EKL29 harbored two virulence genes (*stx_2_* and *eaeA*) while 17 isolates (14.1%) harbored *stx_2_* and *hlyA* virulence determinants, and nine isolates (7.5%) harbored only *stx_2_* virulence gene. Further, in the present research work, five isolates (4.1%) harbored *stx1* and *eaeA* genes, whereas one isolate (0.8%), that is, ERO21 harbored *stx1, stx2*, and *eaeA* genes, while one isolate (0.8%) (EKL3) harbored *stx1, stx2*, and *hlyA* genes. The *stx1* and *stx2* genes were found in nine isolates (7.5%). The distribution of virulence genes was found independent of the location of sample collection.

### 3.4. Biofilm Production

**Figure 2:**
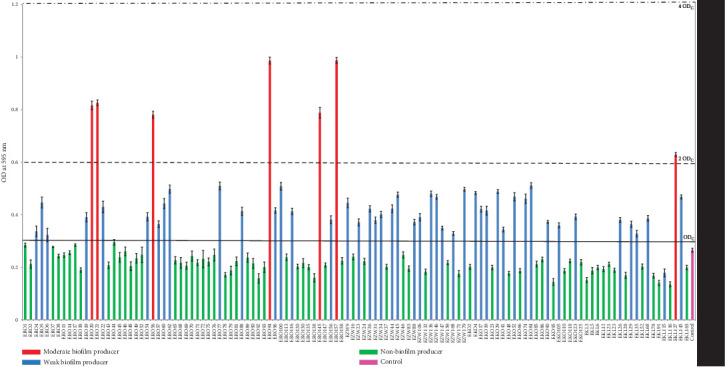
Biofilm formation shown by 120 multiple antibiotic-resistant *E. coli* isolates. The *y* axis represents the median optical density (OD) at 595 nm of three replications, whereas horizontal lines represent the cut-off value for their efficiency in biofilm production. The OD values of samples were compared with the cut-off OD (ODc) to discriminate the efficiency of biofilm production by the isolates. The cut-off OD (ODc) value was calculated as three standard deviations above the negative control. The results were interpreted as: OD < ODc = nonadherent; ODc < OD < (2 ODc) = weak adherent; (2 ODc) < OD < (4 ODc) = moderate adherent; and (4 ODc) < OD = strongly adherent.

In the present study, 120 MAR isolates were screened for biofilm formation. The results showed that 7 strains (ERO20, ERO21, ERO56, ERO94, ERO145, ERO157, and EKL127) were moderate biofilm formers, 45 strains were found to be weak biofilm producers, whereas 68 isolates were nonbiofilm producers (Figure 2). Of the seven moderate biofilm-producing isolates, the isolate ERO157 showed the highest biofilm production. Out of these seven isolates, six were obtained from RO commercial farm, while one was obtained from KL commercial farm. Also, of these seven moderate biofilm producers (Table 4), isolates ERO157 and EKL127 were found to harbor all the four tested virulence genes (*stx1, stx2, hlyA,* and *eae*) whereas isolate ERO21 was found to harbor three tested virulence genes (*stx1, stx2,* and *eae*).

### 3.5. Molecular Typing by PFGE

**Figure 3:**
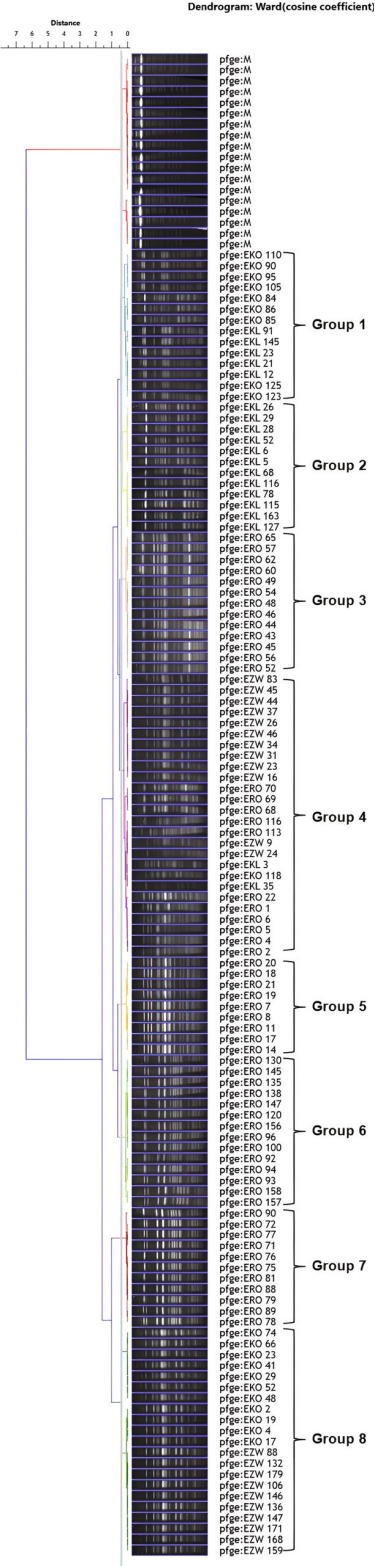
PFGE fingerprinting–based dendrogram showing genetic relation between *E. coli* strains isolated from cattle fecal samples.

In the present study, 120 MAR isolates were clustered into eight groups based on DNA fingerprints generated by PFGE (Figure 3). It was observed that in some groups (Group 1, Group 4, and Group 8) isolates were intermixed within the group irrespective of the location of isolation. In the present study, the majority of groups like Group 2 (KL), Group 3 (RO), Group 5 (RO), Group 6 (RO), and Group 7 (RO) contain isolates that were isolated from the same farm, that is, KL commercial farm, RO commercial farm, but they form separate groups based on the genetic variation among themselves. Group 4 was the largest one, which contained 26 isolates. The huge genetic diversity was observed in the strains isolated from RO commercial farm, as strains isolated from this location formed separate groups (Group 3, Group 5, Group 6, and Group 7) as well as isolates that were also intermixed with other groups. The smallest group was Group 5, which contained nine strains.

## 4. Discussion

*E. coli* belonging to the family *Enterobacteriaceae* is gram-negative bacilli, facultative anaerobe which generally lives as commensal in the gastrointestinal tract of humans and animals [4]. But some strains of *E. coli* evolved as pathogens and cause severe diseases in humans [3]. Food-producing animals, especially cattle, are important carriers of these pathogenic strains [6, 16]. The other way of transfer of these pathogenic strains is through contaminated manure used for growing agricultural crops [4, 6]. Therefore, the present investigation was designed to detect the presence of pathogenic *E. coli* in the fecal samples of cattle. In the present investigation, out of 1566 presumptive isolates, 636 isolates were confirmed as *E. coli* by amplifying genus-specific *uidA* gene. This gene is responsible for the production of *β*-D-glucuronidase which is conserved for the genus *E. coli* [30]. The earlier researchers also targeted the *uidA* gene for the identification of *E. coli* [25, 30–32].

Further, antibacterial susceptibility was done on confirmed 636 strains, and 528 isolates (83.01%) showed resistance to CTX, followed by AMP [414 isolates (65.09%)]. A similar type of prevalence of CTX was reported in the previous studies. Hille et al. [33] reported that 85% and 70% of *E. coli* strains isolated from dairy cattle units and beef cattle units, respectively, showed resistance to CTX. Markland et al. [34] reported 47.4% CTX-resistant bacteria in the cattle fecal samples. Azabo et al. [35] reported that 95% of *E. coli* isolates were resistant to CTX, isolated from cattle fecal samples in Tanzania. CTX is an important drug in human medicine [36]; therefore, the emergence of resistant bacteria to this drug is a cause of concern for public health. Additionally, CTX is the first choice of drug in veterinary medicine used to treat skin wounds and cystitis in dogs and cats. In the present investigation, CTX was found resistant by most strains, which indicates that either natural resistance was persisting in these isolates or that cross-transmission may have transferred the resistance to cattle *E. coli*, as reported previously [37–42] . In the present study, 65.09% of *E. coli* isolates were found resistant to AMP. Dela Pena et al. [43] isolated *E. coli* from different fecal and water samples and found that 43.6% of isolates were resistant to AMP. Hang et al. [44] isolated *E. coli* from fecal samples of young dairy calves and reported that 48.6% of isolates were resistant to AMP. In another study, Goncuoglu et al. [45] found that 63.63% of *E. coli* O157:H7 isolated from cattle fecal samples were resistant to AMP. AMP, which is an old antibiotic, is still widely used in the treatment of infection. AMP is mostly used in human treatment as compared to animals [46]. But due to the misuse of this antibiotic, bacterial cells produce *β*-lactamase, which inactivates the antibiotic, and the genes responsible for *β*-lactamase are exchanged between the bacterial cells using various genetic mechanisms. So, overall, the misuse or inappropriate use of antibiotics in veterinary medicine leads to selective pressure, which generates antibiotic-resistant *E. coli*, and resistant determinants are exchanged between the isolates using various genetic exchange processes [47]. Further, in the present study, 120 (18.86%) isolates were found to be MAR. Previously, researchers also observed varying MAR in *E. coli* strains isolated from cattle fecal samples. Mwenifumbo et al. [48] isolated *E. coli* from calf fecal samples and found that 16 isolates out of 40 (40%) were MAR. Tabaran et al. [49] in their study found that 38% and 68% of *E. coli* were MAR, isolated from the intestinal content during the evisceration step from abattoirs of bovine in France and Romania, respectively. In another study, Jalil et al. [50] found that 93% of their *E. coli* isolates were multidrug-resistant (MDR) isolated from bovine fecal samples. The variation in antibiotic resistance patterns, especially the presence of MAR isolates at different geographical locations, is observed due to the amount and type of uncontrolled usage of antibiotics for both preventative and therapeutic purposes [50]. So, overall, the misuse or inappropriate use of antibiotics leads to selective pressure, which generates antibiotic-resistant *E. coli*, and resistant determinants are exchanged between the isolates using various genetic exchange processes [47]. The presence of different antibiotic-resistant determinants could be present on the same mobile genetic elements (MGEs) like conjugative and integrative elements, insertion sequences, or transposons, which may generate and spread MAR/MDR bacteria globally [51, 52].

Some *E. coli* strains produce virulence genes which are responsible for the strain to be pathogenic. But mere the presence of a virulence gene is not ideal to cause infection and to be pathogenic. To cause disease, bacteria should have some specific combination of virulence genes [53]. Thus, to determine the pathogenic potential of a strain, virulence genes are an ideal target. In the present investigation, a comparatively high occurrence of *stx_2_* virulence gene (48.3%) was found as compared to *stx_1_* (39.1%) and this is a cause of concern as epidemiological studies showed that *E. coli* possessing *stx_2_* is more virulent (associated with HUS) as compared to *stx_1_* [6, 54]. A similar type of observation has been reported [55–57]. In the present study, some strains were found to possess both *stx_1_* and *stx_2_* virulence genes. So, co-expression of both genes may increase the pathogenicity of STEC strains [58]. Additional virulence factors are also required to establish infection, and *eaeA* is an important virulence gene producing intimin present on the locus of enterocyte effacement (LEE) which helps in the attachment of *E. coli* to the intestinal cell, which is a very crucial step in establishing infection [6]. In the current study, 26.6% of isolates harbored the *eaeA* gene. Many isolates harbored both *stx_2_* and *eaeA* genes, which is a cause of concern for public health as this combination may increase the pathogenicity potential of these isolates. Also, the absence of the *eaeA* virulence gene does not rule out the pathogenicity of the isolate as several other adhesion factors are available for attachment [59, 60]. Further, in the present study, the *hlyA* gene was screened in MAR isolates, and 62.5% of isolates harbored this gene. The *hlyA* gene is a plasmid-encoding gene responsible for the production of hemolysin, which causes hemolysis of eukaryotic cells. In the previous studies conducted by different researchers, variable percentages of this gene were reported to persist in the strains isolated from different samples, like fecal and meat [61, 62].

Biofilm, which is a protective polysaccharide covering produced by the bacteria to protect itself from adverse conditions, including penetration of antimicrobial agents into biofilm, not only protects pathogens from adverse conditions but also provides a high amount of infectious dose capable of causing disease [63]. So, the biofilm production characteristics enhance the survival and disease-causing characteristics of pathogens. In the present study, 7 isolates (ERO20, ERO21, ERO56, ERO94, ERO145, ERO157 and EKL127) were found to be moderate biofilm producers, which is a cause of concern, as biofilm production could enhance the resistance of these pathogens against antibiotics. Also, two biofilm-producing isolates, that is, ERO157 and EKL127, were found to possess all four virulence genes, which is a cause of concern, especially in the area where these isolates are circulating.

Further to determine the genetic similarities between the isolates, a PFGE technique was used as this is “gold standard” to know the similarity between isolates at the genetic level. The PFGE technique has high discriminative power [64] which showed DNA fingerprints based on restriction patterns generated by a specific restriction enzyme (*XbaI* was used in the present study). The 120 isolates were grouped into 8 clusters based on genetic prints. Few groups (Group 1, Group 4, and Group 8) were composed of isolates obtained from different locations, which indicated these isolates in the same group have a similar clone. Previously, similar types of results with clonality distribution of *E. coli* were reported [65–67]. In the present study, some strains clustered together in the same group isolated from the same geographical location indicate their unique and similar genetic makeup (Group 2, Group 3, Group 5, Group 6, and Group 7). Also, within the same farm (same geographical location) some strains were clustered into separate groups (Group 3, Group 5, Group 6, and Group 7 which was isolated from RO farm), which indicates clonal diversity of *E. coli* strains persists within the same farm. As cattle are important reservoirs of these strains, they may generate more virulent strains of *E. coli* by exchanging virulence/antibiotic-resistant traits between them. So, these pathogenic isolates could enter into the food chain (animal food or organic manure or contaminated water for irrigation) through cross-contamination.

## 5. Conclusion

In conclusion, the results suggest that cattle in the Northwest region of South Africa carried genetically diverse, virulent, multiple antibiotic-resistant, and biofilm-forming *E. coli* pathotypes. The ability to form biofilms may enhance these pathogens' survival and spread in the environment, potentially increasing the risk of contamination or infection. Additionally, biofilm-forming antimicrobial-resistant isolates could persist in the environment and facilitate the transfer of resistance genes to other bacteria—both pathogenic and nonpathogenic—which may, in turn, pass resistance on to pathogens affecting humans or animals. It is recommended that farm owners are routinely sensitized about hygienic protocols, and the judicious use of antibiotics on the farm should be done under the guidance of a veterinary doctor so as to prevent the infection caused by pathogenic *E. coli*. To ensure the safety of people, regular surveillance of pathogens such as *E. coli* on the cattle farm should be encouraged, as these farms could be an important source of antibiotic resistance dissemination. The present study provides baseline information regarding the persistence of antibiotic-resistant, virulent *E. coli* in cattle feces, and this information could be utilized by health practitioners for the treatment of patients infected by *E. coli* in the studied area. Further studies are required to understand the transmission dynamics of virulence and antibiotic-resistant genes between environment–cattle–human intersections so that appropriate strategies can be adopted to prevent the cross transmission of these genes.

## Figures and Tables

**Table 1:** Different commercial farms from where fecal samples were collected.

**Location/farms**	**Code of the farms**	**No. of samples collected**	**Location**
Rooigrond commercial farm	RO	70	Mafikeng
Zwartfontein commercial farm	ZW	64	~150 km from Mafikeng
Koster commercial farm (Lichtenburg)	KO	59	~69 km from Mafikeng
Klippan commercial farm	KL	68	~70 km from Mafikeng
Total		261	

**Table 2:** The details of primer sequences and conditions of PCR used for identification and profiling of virulence genes.

**Primer**	**Sequence (5**⁣′**-3**⁣′**)**	**Target gene**	**Amplicon size (bp)**	**PCR conditions and cycles**	**Reference**
uidA F	CTGGTATCAGCGCGAAGTCT	*uidA*	556	Initial denaturation at 95°C for 10 min, 35 cycles at 95°C for 45 s, 30 s at 59°C, 1 min 30 s at 72°C; 1 cycle of 10 min at 72°C	[25]
uidA R	AGCGGGTAGATATCACACTC				
stx1 F	ATAAATCGCCATTCGTTGACTAC	*stx_1_*	180	Initial denaturation at 95°C for 1 min, 35 cycles of 95°C for 1 min, 62.5°C for 2 min, 72°C for 1 min 30 sand final elongation at 72°C for 5 min	[26]
stx1 R	AGAACGCCCACTGAGATCATC				
stx2 F	GGCACTGTCTGAAACTGCTCC	*stx_2_*	255		
stx2 R	TCGCCAGTTATCTGACATTCTG				
hlyA F	GCATCATCAAGCGTACGTTCC	*hlyA*	534		
hlyA R	AATGAGCCAAGCTGGTTAAGCT				
eaeA F	GACCCGGCACAAGCATAAGC	*eaeA*	384	Initial denaturation at 95°C for 1 min, 35 cycles of 95°C for 1 min, 62°C for 2 min, 72°C for 1 min 30 s and final elongation at 72°C for 5 min	
eaeA R	CCACCTGCAGCAACAAGAGG				

**Table 3:** The virulence genes profile of multiple antibiotic-resistant strains isolated from cattle fecal samples.

**Farm**	**Isolates positive for virulence gene**					
	***stx1***	***stx2***	***hlyA***	***eae***	***stx1/stx2***	***stx1/stx2/hlyA/eae***
Rooigrond commercial farm (RO) *n* = 58	25	28	35	17	5	5
Zwartfontein commercial farm (ZO) *n* = 22	4	9	15	4	0	1
Koster commercial farm (KO) *n* = 21	9	11	14	6	2	3
Klippan commercial farm (KL) *n* = 19	7	13	11	5	1	2
% prevalence of gene/genes	37.5	50.8	62.5	26.6	6.6	9.1

**Table 4:** The antibiotic resistance pattern and virulence genes profile of seven moderate biofilm-producing isolates.

**Traits**		**Isolates**						
		**ERO20**	**ERO21**	**ERO56**	**ERO94**	**ERO145**	**ERO157**	**EKL127**
Antibiotic susceptibility	AMP	R	R	R	R	R	R	R
	ETP	R	S	I	R	I	R	S
	CTX	R	R	R	R	R	R	R
	CIP	R	I	I	R	I	I	I
	GM	I	S	S	R	S	I	S
	TE	R	R	R	I	R	R	R
	NOR	S	S	S	S	S	S	S

Virulence genes	*stx1*	−	+	+	−	+	+	+
	*stx2*	+	+	−	+	+	+	+
	*hlyA*	−	−	+	+	−	+	+
	*eae*	−	+	−	−	−	+	+

*Note:* + means present, and − means absent.

Abbreviations: AMP, ampicillin; CIP, chloramphenicol; CTX, cefotaxime; ETP, ertapenem; GM, gentamicin; I, intermediate resistant; NOR, norfloxacin; R, resistant; S, susceptible; TE, tetracycline.

## Data Availability

The data that support the findings of this study are available from the corresponding author upon reasonable request.
